# Uncovering unmet needs in healthcare teams: a cross-sectional study on the second victim phenomenon

**DOI:** 10.1186/s13584-026-00764-1

**Published:** 2026-05-20

**Authors:** Shani Fisher, Ranit Djerassi, Yulia Gendler, Avinoam Pirogovsky, Jacob Dreiher

**Affiliations:** 1 Emek Medical Center, Afula, Israel; 2 Clalit Health Services, Tel Aviv, Israel; 3 Nursing Department, Ariel University, Ariel, Israel

**Keywords:** Second victim, Healthcare teams, Medical errors, Support systems, Survey study

## Abstract

**Background:**

Healthcare teams encounter adverse events and medical errors, and inadequate awareness and support can leave these teams vulnerable as “second victims” (SV). The objectives of our study were to assess healthcare professionals’ understanding of the SV concept, evaluate the existing support systems in place, and identify the types of support mechanisms they consider most desirable.

**Methods:**

In February 2024, the SV Experience and Support online survey was administered to healthcare workers in hospitals owned by Clalit Health Services, Israel. The survey focused on emotional responses following involvement in medical errors, perceived support from supervisors, and the availability of organizational resources. Statistical analysis was performed using SPSS statistical software. A Likert scale was used, where 1 represented “strongly disagree” and 6 represented “strongly agree.”

**Results:**

The survey was sent to 6,645 physicians and 11,855 nurses. A total of 483 physicians and 1,359 nurses participated in the survey (7.2% and 11.4% response rate, respectively). Both physicians and nurses reported high emotional stress (Mean scores: 4.89 ± 1.17 and 5.16 ± 1.06, respectively, *p* < 0.001) and embarrassment (Mean scores: 4.76 ± 1.16 and 4.94 ± 1.22, respectively, *p* < 0.001) following errors, with a notable desire for peer support (51.6%). Sleep disturbances also highlighted the adverse events’ somatic burden and impact (Mean scores: 3.96 ± 1.49 and 4.22 ± 1.50, respectively, *p* = 0.002). The most desired support path was having a colleague to discuss the event (*p* < 0.001).

**Conclusions:**

Healthcare workers face a significant risk of becoming SV after unexpected medical events, and peer support is preferred. Program directors should therefore prioritize structured peer support initiatives, alongside robust formal systems and open communication, in accordance with workers’ preferences. Addressing gaps in resource awareness and refining managerial approaches is essential.

**Supplementary Information:**

The online version contains supplementary material available at 10.1186/s13584-026-00764-1.

## Background

In high-income countries, approximately one in ten patients experience an adverse event while receiving hospital care. Worryingly, in low- and middle-income countries, estimates suggest that up to one in four patients is harmed. These unsafe hospital practices result in an estimated 134 million adverse events annually, contributing to approximately 2.6 million deaths each year [1].

In 2000, Albert Wu introduced the concept of the Second Victim (SV), referring to the emotional suffering experienced by healthcare providers after a medical error, even when they are not physically harmed. The SV experience can affect a variety of professionals, including nurses, physicians, pharmacists, and other healthcare workers, largely due to the constraints of hospital hierarchies and limited control over clinical outcomes [2]. Research indicates that the prevalence of SV responses following an adverse event range from 10.4% to 43.3% [3]. These experiences manifest through a complex mix of emotional responses, such as feelings of guilt, anger, psychological distress, depression, and fear, as well as anger and frustration. Additionally, physical effects like burnout are commonly reported among those impacted by SV [4]. Among these complex reactions, it is essential to acknowledge the moral dilemmas related to professional responsibility. Healthcare workers often grapple with thoughts of leaving the profession due to feelings of guilt, shame, and self-doubt following SV’s experiences [4–7].

SV support programs aim to accelerate the recovery of healthcare providers after involvement in an adverse event while addressing their emotional and psychological needs. These programs are vital for fostering a culture of safety by encouraging open communication, minimizing the fear of blame, and building both individual and organizational resilience. Through immediate assistance, peer support, and strategies for coping and resilience-building, SV programs not only mitigate the short-term emotional impact of adverse events but also strengthen long-term adaptive capacity and safety within healthcare organizations [8]. The methods used in SV support programs are varied, incorporating peer support, structured interventions, and evidence-based psychological frameworks to address the emotional and professional needs of affected healthcare providers. Most support plans rely on peer-based models, where trained colleagues provide confidential emotional and practical assistance, either in one-on-one settings or group support formats. These peer supporters undergo specific training and, in some cases, regular debriefings and continuous training sessions to ensure they are well-prepared to provide support [9, 10].

Current research reveals a critical disconnect: major institutions such as Clalit Health Services have established support resources, yet there remains a significant gap in staff awareness of and utilization of these tools. This disconnect suggests that existing policies may be failing to reach those in need. Consequently, there is an urgent imperative to identify the barriers to resource scaling as well as the specific, unmet support preferences of frontline staff to ensure organizational resilience.

Although the SV phenomenon is widely recognized, effective strategies and mechanisms to support healthcare professionals impacted by it are still lacking. This study addresses this gap by exploring healthcare teams’ attitudes and perceptions related to the SV experience. The primary objective is to evaluate healthcare professionals’ awareness of the SV concept and assess the current support mechanisms. Furthermore, the research investigated how these factors vary across different healthcare populations. The study investigates the disparity between available organizational support and its actual utilization among healthcare teams. Specifically, we aim to evaluate healthcare professionals’ awareness of the SV concept, assess the perceived utility of current support mechanisms, and identify the specific pathways, such as peer-to-peer versus formal managerial support, that staff consider most effective. By uncovering these unmet needs, this study seeks to provide a roadmap for more effective policy implementation and enhanced mental well-being for healthcare professionals and to enhance the effectiveness of support systems and promote the mental well-being of healthcare professionals.

## Methods

### Research design

This study utilized an anonymous online cross-sectional survey distributed via organizational email to all physicians and nursing teams within Clalit Health Services, the largest healthcare provider organization in Israel that serves about 52% of Israel’s population, including nearly two-thirds of the country’s older adult population.

### Participants

The participants in this study were healthcare professionals (physicians and nurses) from 14 hospitals owned by Clalit.

### Data collection

An online survey was distributed through the organizational email system twice weekly for one month during February 2024. The survey included 18 questions in total: ten statements with a Likert scale, where 1 represented “Strongly Disagree” and 6 represented “Strongly Agree” and eight additional questions (see Supplementary Table 1). The survey was adapted from the Medically Induced Trauma Support Services’ “Staff Support Survey Tool” [11]. The survey asked about a) the definition of SV, b) emotional responses following involvement in medical errors , c) thinking about leaving work, d) perceived support from supervisors, and e) the availability and use of organizational resources; f) preferred coping mechanisms, as well as g) their profession, years of professional experience and having a managerial role. The survey was previously used in Clalit and was recognized as having face validity. In addition, reliability was assessed in the present study using exploratory factor analysis and Cronbach’s alpha.

### Data analysis

Data were analyzed using SPSS (version 29). Continuous and ordinal variables were summarized using descriptive methods, as the data were ordinal. The Chi-Square test was applied to examine relationships between categorical variables. Additionally, the Kruskal-Wallis rank sum test and Pearson’s Chi-squared test were used to assess differences between groups and associations between categorical variables, respectively. Factor analysis was conducted to explore the underlying structure of the data and identify latent factors influencing the observed variables. This analysis was performed on two categories: participants’ perspectives on SV support and symptoms experienced as an SV. The reliability of the identified factors was assessed using Feldt’s alpha with 95% confidence intervals.

## Results

A total of 6,645 physicians and 11,855 nurses were invited to take part in the survey, and a total of 483 physicians (7.2% response rate) and 1,359 nurses (11.4% response rate) completed the survey. The demographic characteristics of participants are demonstrated in Table 1.

**Table 1:** Demographic characteristics of participants in the survey

Year of professional experience	Physicians (*N* = 483)	Nurses (*N* = 1359)	Total (*N* = 1842)
Up to 1 year	32 (8.7%)	38 (3.6%)	70 (4.9%)
1–5 years	65 (17.6%)	154 (14.5%)	219 (15.3%)
6–10 years	47 (12.7%)	138 (13.0%)	185 (12.9%)
11–15 years	63 (17.1%)	129 (12.2%)	192 (13.4%)
Over 15 years	162 (43.9%)	602 (56.7%)	764 (53.4%)
**Managerial role**	123 (33.5%)	255 (24.1%)	378 (26.6%)

### Understanding of the “second victim” (SV) concept

The majority of participants (75.8%) correctly identified the SV as a healthcare professional involved in an adverse event. A slightly larger proportion of physicians (79.8%) correctly identified the concept, compared with nurses (74.3%). However, 17.5% of respondents were unfamiliar with the term, indicating a gap in education and awareness.

### Symptoms of participants as “second victim” (SV)

Healthcare professionals experience significant emotional distress following their involvement in adverse events. In most statements, scores were higher for nurses than for physicians, reflecting significantly higher levels of stress in nurses (Table 2). These negative emotions included feeling stressed after discovering an error, feelings of remorse, embarrassment, and sleep disturbances.

As for the impact of peer and managerial support, peer support was identified as a crucial coping mechanism. A majority of respondents (52%) reported that “conversations with colleagues following adverse events helped alleviate” their stress. Perceptions of managerial support showed more variability. Interestingly, while most participants felt appropriately treated by their managers after making an error, nurses significantly reported feeling blamed by their supervisors compared to physicians (*p* < 0.001). A small but notable proportion of respondents indicated that stress from errors led them to consider leaving their profession, with a median score of 2.00 for both sectors (*p* = 0.204). This suggests that while not universal, the cumulative impact of errors could contribute to professional dissatisfaction and potential turnover (Table 2). These findings suggest a disparity in how support is perceived and delivered across professional roles.

**Table 2:** Symptoms of participants as second victim (SV)

Statement	Physicians			Nurses			Total			*P* value*
	Mean (SD)	Median	*N*	Mean (SD)	Median	*N*	Mean (SD)	Median	*N*	
I was very stressed after discovering the error	4.89 (1.17)	5.00	396	5.16 (1.06)	5.00	1130	5.09 (1.09)	5.0	1526	< 0.001
I had remorse for my involvement in the event	4.99 (1.13)	5.0	393	5.09 (1.14)	5.0	1121	5.07 (1.14)	5.0	1514	0.029
I felt embarrassed after being involved in such events	4.76 (1.16)	5.0	390	4.94 (1.22)	5.0	1116	4.89 (1.20)	5.0	1506	< 0.001
Involvement in a significant event caused me to have insomnia	3.96 (1.49)	4.0	390	4.22 (1.50)	4.0	1113	4.15 (1.50)	4.0	1503	0.002
I considered leaving my job	2.5 (0.7)	1.8	380	2.5 (2.1)	2.0	1084	2.6 (1.4)	1.0	1463	0.204
A conversation with a colleague helped me after such an event	4.56 (1.23)	5.0	390	4.54 (1.23)	5.0	1106	4.54 (1.23)	5.0	1496	0.706
I feel that my manager treats me appropriately after I make an error	4.62 (1.35)	5.0	387	4.39 (1.40)	5.0	1097	4.45 (1.39)	5.0	1484	0.003
My manager blames the workers	2.60 (1.32)	2.0	387	2.94 (1.47)	3.0	1097	2.85 (1.44)	3.0	1484	< 0.001

*Kruskal-Wallis rank sum test

### Awareness and utilization of institutional support systems

The survey highlighted gaps in the availability and awareness of institutional support systems, with only 37.3% of respondents aware of formal support for SV. Physicians were slightly less aware than nurses. Despite available support, only 15.6% utilized it. The majority (53.5%) reported that no support was offered, while 26.3% felt it was unnecessary (Table 3). These findings underscore the need for proactive outreach and accessible, effective support resources.

**Table 3:** Participants’ attitudes towards organizations’ support

Statement	Physicians			Nurses			Total			*P* value*
	Mean (SD)	Median	*N*	Mean (SD)	Median	*N*	Mean (SD)	Median	*N*	
The hospital leaders where I work understand that healthcare professionals involved in an error may need help to process and overcome the incident	3.87 (1.42)	4.0	378	3.99 (1.40)	4.0	1091	3.96 (1.41)	4.0	1469	0.186*
My organization offers various resources to help recover from the impact of being involved in an error	3.46 (1.41)	3.0	371	3.53 (1.38)	4.0	1075	3.51 (1.39)	4.0	1446	0.448*
The right definition of the term “Second Victim.”	296 (79.8%)		374	785(74.3%)		1057	1081 (75.8%)		1427	0.069
There is a Support system for SV	129 (35.1%)		367	402 (38%)		1056	531 (37.3%)		1424	0.347

*Kruskal-Wallis rank sum test

### Desired support pathways

When asked about preferred support, the most desired option was having a colleague to discuss the event (*p* < 0.001) with physicians more likely to choose this option (*p* < 0.001, Table 4). This is in accordance with participants’ past experiences, where 52% of respondents also reported that conversations with colleagues following adverse events helped alleviate their stress. Of note, 26% of participants favored a quiet space for recovery, with nurses showing a slightly higher preference. In addition, physicians were slightly more likely to self-refer for help, while about two-thirds of participants did not seek any help. Of those who were offered help, nurses were more likely to “feel it was not needed” (Table 4).

**Table 4:** Desired and actual support pathways

Support pathways	Physicians (*N* = 483)	Nurse (*N* = 1359)	Total (*N* = 1842)	*p*-value
Immediate time-out from the unit	111 (23.0%)	289 (21.3%)	400 (21.7%)	0.432
Designated quiet space to recover	110 (22.8%)	369 (27.2%)	479 (26.0%)	0.060
Colleague to discuss the event	282 (58.4%)	668 (49.2%)	950 (51.6%)	< 0.001
Free professional counseling outside the hospital	128 (26.5%)	325 (23.9%)	453 (24.6%)	0.257
Discussion with my manager	209 (43.3%)	508 (37.4%)	717 (38.9%)	0.023
Time with a hospital consultant	160 (33.1%)	94 (29.0%)	554 (30.1%)	0.089
24/7 contact with a support person	80 (16.6%)	355 (26.1%)	435 (23.6%)	0.398
If you were involved in a serious event/error with a severe outcome for a patient, was support offered to you by the hospital?				0.071
I was referred for help	61 (20.5%)	166 (24.3)	227 (23.2%)	
I self-referred for help	44 (14.8%)	69 (10.1%)	113 (11.5%)	
I did not seek help	192 (64.6%)	448 (65.5%)	640 (65.3%)	
If support was offered, did you make use of this opportunity?				0.046
Yes	53 (18.3%)	146 (21.0%)	199 (20.2%)	
I did not feel it was needed	64 (22.1%)	195 (28.0%)	359 (26.3%)	
I was not offered help	172 (59.5%)	355 (51.0%)	527 (53.5%)	

### Reliability and factor analysis

Factor analysis was performed. Kaiser-Meyer-Olkin (KMO) test was 0.732 (adequate for factor analysis) with a Bartlet’s test of sphericity χ^2^ = 4505, df = 55, *p* < 0.001. Exploratory factor analysis identified a three-factor structure explaining the questionnaire responses: a) psychological distress following medical error (Eigenvalue: 2.937, Percent variance explained: 25.1%, Cronbach’s alpha = 0.83) b) perceived organizational support (Eigenvalue: 2.358, Percent variance explained: 14.7%, cumulative variance: 39.8%, Cronbach’s alpha = 0.65) and c) Supervisory support (Eigenvalue: 1.143, Percent variance explained: 12.2%, cumulative variance: 52.0%, Cronbach’s alpha = 0.66). The item “I considered leaving my job” did not load well with other factors, probably indicating a different construct (burnout or turnover intention).

## Discussion

Survey results indicated that both physicians and nurses reported high stress levels, with a similar prevalence of guilt and embarrassment following errors, some sleep disturbances, and a small but notable proportion considering leaving their profession due to stress from errors. These findings align with previous studies, which have also reported both somatic and psychological symptoms following involvement in adverse events [5–6, 9–14]. Survey results show differences between physicians and nurses in SV. Both groups experienced high stress, but nurses felt more embarrassed and had more sleep disturbances. Physicians reported better managerial support, while nurses felt more blamed. Physicians preferred peer discussions, and nurses favored quiet spaces. Despite slightly higher awareness of support systems among nurses, utilization was low in both groups. Physicians, more likely to hold managerial roles, may perceive blame and support differently. These findings suggest the need for tailored support strategies for both groups. Previous studies provide limited information, but a meta-analysis on SV syndrome in intensive care unit healthcare workers found that the prevalence differences between occupations may not be significant [12].

Survey results indicated that only 37% knew about the support system offered. The unfamiliarity with support systems is a major gap [13]. The most desirable support pathway was the need for “a colleague to discuss the event” as 75% in both sectors replay it. In Clalit, the leading support system is peer support, provided confidently by authorized team members. Certification for this role is achieved through a dedicated course led by a professional, typically a psychologist. However, most participants preferred discussing events with colleagues, with physicians showing a stronger preference. This result can be explained by the gap in familiarity with existing support pathways. Leadership plays a pivotal role in shaping these perceptions. Managers who actively provide support and foster a blame-free environment can help reduce the psychological burden on SV. To evidence, discussing incidents with managers was viewed positively by 43.3% of physicians and 37.4% of nurses, demonstrating that leadership engagement is valued but underutilized. A systematic review exploring coping strategies for the SV phenomenon identified both personal and organizational approaches. Highly valued strategies included peer support and learning from adverse events. Organizational strategies emphasized SV support programs with rapid-response peer teams [14]. To respond to these concerns, international guidelines urge institutions to implement systems that support clinicians following adverse events [15]. Regrettably, organizations often fail to adequately address the needs of their clinicians. A large-scale survey conducted in the U.S. and Canada found that 90% of physicians reported that hospitals and healthcare organizations do not provide sufficient support for managing the stress associated with medical errors [16].

The pathways of support for healthcare professionals affected by adverse events involve several core psychological interventions and theoretical models aimed at addressing emotional distress and promoting recovery. Psychological First Aid and Critical Incident Stress Management (CISM) are two key interventions, widely used to provide immediate crisis response and emotional relief. These interventions aim to reduce acute psychological distress and ensure access to continued care if needed [17]. Additionally, Scott’s Three-Tiered Interventional Model of Second Victim Support and the Just Culture Model provide a framework for these support strategies. The former emphasizes emotional recovery for healthcare professionals, while the latter seeks to reduce systemic blame and encourage a culture of safety [18]. Another influential framework is the Social Resilience model, which focuses on building resilience among individuals to promote long-term organizational resilience. This model emphasizes the importance of proactive strategies, such as peer identification, self-reporting, and systematic reviews of adverse events, to identify individuals affected early [19]. A systematic review of SV support resources identifies 12 formalized, institution-based programs, mostly in the United States, with such systems still scarce globally. The findings emphasize the need for significant investment in establishing SV support programs offering immediate, medium-, and long-term support, promoting existing resources, and monitoring the well-being of peer supporters [9].

The prevalence of sleep disturbances after an event among staff underscores the significant somatic burden of the SV phenomenon. Our findings showed that nurses reported significantly higher levels of insomnia compared to physicians, which indicates a profound physiological manifestation of clinical distress. These sleep disruptions and insomnia may represent a failure of the recovery process, where the emotional toll of an error prevents the physiological rest necessary for stable cognitive function. Recent research supports this pattern, as they identified that nearly 20% of their respective cohorts had insomnia or sleep disturbances following a clinical complication. These identical data points emphasize that somatic symptoms are a universal response to clinical trauma and can compromise professional longevity as well as patient safety [20, 21].

Our data demonstrates that while knowledge regarding the term “second victim” is well established through education and formal orientation, the actual utilization of support services remains relatively low. Despite the existence of these organizational offers, staff members frequently choose not to engage with formal support systems. This disparity suggests that theoretical familiarity with the concept does not automatically translate into the practical adoption of institutional resources. Studies show that even when staff members can define the SV phenomenon and acknowledge that support structures exist, they often express a preference to cope on their own. Many employees feel uncomfortable approaching peers for help or lack practical knowledge regarding how the support process actually functions. Systematic reviews describe this phenomenon as a structural and cultural gap where formal programs and awareness campaigns do not automatically translate into active utilization.

This gap is likely driven by blame-oriented cultures and professional stigma, alongside a general reluctance to ask for help and concerns regarding the confidentiality of available resources. At the same time, simply knowing that an organized support process exists can function as a psychological safety net and enhance the overall perception of organizational support. Closing the disconnect between conceptual awareness and real-world use requires high visibility and easy access to services. Furthermore, leadership must focus on tailoring interventions to specific staff needs and performing active work to reduce the stigma surrounding help-seeking after adverse events [10, 22].

### Policy implications and recommendations

Both the Israel Ministry of Health [23] and the Joint Commission International (JCI) [24] mandate that healthcare organizations implement formal SV support policies. In alignment with the Israel Ministry of Health (MOH) regulations, healthcare organizations are required to implement structured support mechanisms for staff members following adverse events, emphasizing a safety culture that prioritizes systemic learning over individual blame [23]. According to standard GLD.04.01 of the JCI (2024) standards for hospitals, leadership must establish a process for responding to sentinel events that includes support for staff members involved [24].

While much of the literature focuses on the immediate clinicians involved in an error, the concept of the “third victim” extends to the healthcare organization itself. This group includes patient safety officers, risk managers, investigators, and administrative leaders. These individuals are frequently exposed to secondary trauma as they manage the systemic fallout of adverse events, such as intensive investigations, sensitive disclosures, legal risks, and reputational threats. Unlike the SV, the third victim’s distress often stems from cumulative exposure rather than a single event, resulting in moral injury, hypervigilance, and role conflicts. If left unaddressed, this indirect trauma can erode the institutional safety culture, increase turnover in critical leadership roles, and hinder the transparency necessary for incident reporting [25–27].

Education regarding the SV phenomenon should not be delayed until an adverse event occurs. Rather, it should be integrated into the undergraduate curriculum of medical, nursing, and allied health professionals’ education, to build foundational resilience. This should be further reinforced in graduate training. Training must be followed by mandatory procedural orientation during the transition to clinical practice, where the specific roles of the Patient Safety and Risk Management Unit are clarified. Finally, ongoing leadership training is essential to ensure that peer-support protocols are active and that the organizational culture supports the ‘third victims’ responsible for managing clinical fallout.

The Patient Safety and Risk Management Unit should serve as the leading authority regarding support for the SV. It needs to hold primary responsibility for the immediate coordination of assistance to staff members involved in adverse events. Beyond providing this immediate intervention, the unit should be tasked with additional roles. These roles include the longitudinal responsibility of educating the staff and maintaining support for the organizational safety culture.

This support system should be executed through a dedicated Staff Support Team, composed of interdisciplinary members who went thorough specialized training in psychological first aid and peer-support techniques. It is recommended that this team be specifically designed to meet the needs of staff members who proactively seek assistance or are identified as being at risk following a sentinel event. By maintaining a cohort of specially trained responders, the organization will ensure that the support provided is both clinically competent and sensitive to the unique pressures of the healthcare environment. If needed, more intensive support must be provided.

To mitigate risks regarding the third victim, the organization must implement a support structure that includes these indirect roles within its safety policies. In routine cases, immediate peer support should be standard for all staff members affected by the event, while moderate distress warrants will require professional assistance. For third victims, the Patient Safety and Risk Management Unit must ensure that support is framed as a standard operational necessity for all high-risk roles. Leaders must be trained to recognize the signs of indirect trauma within their own ranks to foster a genuine no-blame culture. If needed, both should consider a group intervention as well as an individual one [26, 27].

Healthcare organizations should monitor the knowledge and attitudes of staff regarding the second and third victim phenomena through periodic surveys such as the one reported in the present study. Of note, the current safety culture questionnaire employed by the MOH (and based on the Agency for Health Research and Quality questionnaire [28] includes one question regarding support for the SV. We recommend expanding this questionnaire to include additional questions regarding support for SVs, their actual utilization, and satisfaction with the support provided.

### Limitations

The study is limited by its cross-sectional design and reliance on self-reported data. A non-response bias is an option due to the relatively low response rate, lower for physicians (7.2%) than for nurses (11.4%). This raises the risk of inflated SV prevalence, as responders might be more emotionally invested and might over-represent workers experiencing the SV and/or less favorable outcome. Therefore, results should be interpreted with caution, avoiding over-generalization to Clalit’s full workforce). Study participants were workers in Clalit-owned hospitals. Knowledge and attitudes towards the SV phenomenon in the community were not explored. Furthermore, the study results may have limited generalizability because they focus solely on Clalit Health Services. While Clalit is a major healthcare provider, its internal culture does not necessarily represent the entire Israeli healthcare system. Additionally, the Israeli healthcare system does not represent the global context due to unique cultural and legal norms. Differences in professional hierarchy or liability frameworks across countries mean that these findings should be generalized to international healthcare systems with caution. Future research could explore longitudinal effects and the effectiveness of specific support mechanisms. Further research is needed to examine the long-term psychological effects of the SV phenomenon and to evaluate the efficacy of different support programs.

## Conclusion

This study provides valuable insights into the emotional and psychological impacts of medical errors on healthcare professionals. Despite some existing support systems, the findings reveal that more effective and comprehensive measures are needed to assist healthcare professionals who experience the SV phenomenon. This research highlights the necessity for healthcare organizations to enhance support systems and raise awareness to mitigate the emotional impact on healthcare workers. Furthermore, organizational leadership must foster a safety culture based on principles of no shame and no blame to reduce the likelihood of teams becoming SVs following adverse events or errors. These findings could inform future policy changes to improve both staff well-being and patient care.

## Supplementary Material

Supplementary Material 1.

## Data Availability

The data that support the findings of this study are available from Clalit Health Services, but restrictions apply to the availability of these data, which were used under license for the current study, and so are not publicly available. Data are, however, available from the authors upon reasonable request and with permission of Clalit Health Services.

## Abbreviation

SV: Second victim
