# Supplementary material for: Uncovering unmet needs in healthcare teams: a cross-sectional study on the second victim phenomenon
Source: Isr J Health Policy Res. 2026 May 20;15:19. doi: 10.1186/s13584-026-00764-1 (PMC13188354; doi:10.1186/s13584-026-00764-1)
Supplement: Supplementary file 1 — Supplementary Material 1. [file 13584_2026_764_MOESM1_ESM.docx]

**Supplementary Table 1: Study Questionnaire**

**Dear Colleagues,**

We all make errors at least once, and sometimes these errors result in harm to a patient. Those involved in such errors experience emotional turmoil, including anxiety, shame, anger, and guilt-a response similar to post-traumatic stress. It is unreasonable to expect a staff member who has made an error to continue working without addressing the emotional impact of the error.

We aim to assess whether organizational culture has changed since the previous survey and whether the topic has been integrated into all hospitals' work programs.

We kindly ask for your help: Please answer the attached short, anonymous questionnaire candidly.

We appreciate your cooperation,
*The Patient Safety and Risk Management Department, Clalit Health Services*

**Survey Questions**

**Please indicate your level of agreement with each of the following statements:**

*(1 = Strongly Disagree, 6 = Strongly Agree)*

| **Statement** | **1** | **2** | **3** | **4** | **5** | **6** |
| --- | --- | --- | --- | --- | --- | --- |
| 1. I felt very stressed after discovering the error. | ☐ | ☐ | ☐ | ☐ | ☐ | ☐ |
| 2. I experienced feelings of guilt related to my involvement in the incident. | ☐ | ☐ | ☐ | ☐ | ☐ | ☐ |
| 3. I felt embarrassed after being involved in incidents of this nature. | ☐ | ☐ | ☐ | ☐ | ☐ | ☐ |
| 4. Involvement in a serious event caused me to experience sleep disturbances. | ☐ | ☐ | ☐ | ☐ | ☐ | ☐ |
| 5. A conversation with a colleague following an event like this was helpful to me. | ☐ | ☐ | ☐ | ☐ | ☐ | ☐ |
| 6. I feel that my supervisor treats me appropriately after I make an error. | ☐ | ☐ | ☐ | ☐ | ☐ | ☐ |
| 7. My supervisor blames people. | ☐ | ☐ | ☐ | ☐ | ☐ | ☐ |
| 8. The hospital I work at understands that healthcare providers involved in errors may need assistance to process and cope with their impact. | ☐ | ☐ | ☐ | ☐ | ☐ | ☐ |
| 9. My organization offers a variety of resources to assist with recovery from the effects of involvement in an error. | ☐ | ☐ | ☐ | ☐ | ☐ | ☐ |
| 10. The pressure from errors makes me want to leave my profession. | ☐ | ☐ | ☐ | ☐ | ☐ | ☐ |

**11. Which of the following mechanisms would you prefer to have access to following involvement in an error? *(Select multiple options if applicable)***

☐ The ability to take immediate time off from the department for a short period.

☐ A designated quiet space to recover and "regroup" following such an event.

☐ A trusted colleague with whom I can discuss the event.

☐ A support program offering free professional counseling outside the hospital.

☐ A conversation/discussion with my supervisor about the event.

☐ The option to schedule time with a counselor/supporter at my hospital to discuss the event.

☐ The option to contact a professional available 24/7 to discuss the potential impact of the event on me and my professional future.

☐ Other (please specify): _______________________________

**12. What does the term "Second Victim" mean to you? *(Select one option)***

☐ A staff member involved in an event with a severe outcome.

☐ The family members of the patient who was harmed in a serious event.

☐ An event involving multiple patients who were harmed.

☐ I am not familiar with this term.

**13. Does your hospital have support for "Second Victim" situations? *(Select one option)***

☐ Yes- there is a support system.

☐ No, I am not aware of such a system.

**14. If you were involved in a serious event/error with a severe outcome for a patient, was support offered to you by the hospital? *(Select one option)***

☐ I was offered to contact a designated contact person at my hospital trained on the subject.

☐ I was offered to contact a designated person trained on the subject from another hospital.

☐ I was offered to contact my colleagues.

☐ I was offered to privately contact a professional counselor.

☐ No support was offered to me.

**15. If support was offered, did you make use of this opportunity? *(Select one option)***

☐ Yes- I spoke with the contact person I was referred to.

☐ I used private professionals for assistance.

☐ I didn’t feel the need for support.

☐ No support was offered to me.

**Additional Information**

**16. Years of Professional Experience:**

☐ Up to 1 year

☐ 1–5 years

☐ 6–10 years

☐ 10–15 years

☐ More than 15 years

**17. Your role in the department/clinic/unit:**

☐ Managerial position

☐ Non-managerial position

**18. occupation:**

☐ Physician

☐ Nurse
